# Environmental specificity of karst cave habitats evidenced by diverse symbiotic bacteria in Opiliones

**DOI:** 10.1186/s12862-024-02248-9

**Published:** 2024-05-08

**Authors:** Likun Zhao, Ruoyi Xiao, Shanfeng Zhang, Chao Zhang, Feng Zhang

**Affiliations:** 1 School of Life Sciences, Institute of Life Science and Green Development, Hebei University, Baoding, 071002 P.R. China; 2 The Key Laboratory of Microbial Diversity Research and Application of Hebei Province, Baoding, 071002 P. R. China; 3 The Key Laboratory of Zoological Systematics and Application of Hebei Province, Baoding, 071002 P. R. China

**Keywords:** Symbiotic bacteria, Opiliones, Karst cave, Ecology

## Abstract

**Background:**

Karst caves serve as natural laboratories, providing organisms with extreme and constant conditions that promote isolation, resulting in a genetic relationship and living environment that is significantly different from those outside the cave. However, research on cave creatures, especially Opiliones, remains scarce, with most studies focused on water, soil, and cave sediments.

**Results:**

The structure of symbiotic bacteria in different caves were compared, revealing significant differences. Based on the alpha and beta diversity, symbiotic bacteria abundance and diversity in the cave were similar, but the structure of symbiotic bacteria differed inside and outside the cave. Microorganisms in the cave play an important role in material cycling and energy flow, particularly in the nitrogen cycle. Although microbial diversity varies inside and outside the cave, Opiliones in Beijing caves and Hainan Island exhibited a strong similarity, indicating that the two environments share commonalities.

**Conclusions:**

The karst cave environment possesses high microbial diversity and there are noticeable differences among different caves. Different habitats lead to significant differences in the symbiotic bacteria in Opiliones inside and outside the cave, and cave microorganisms have made efforts to adapt to extreme environments. The similarity in symbiotic bacteria community structure suggests a potential similarity in host environments, providing an explanation for the appearance of *Sinonychia martensi* in caves in the north.

**Supplementary Information:**

The online version contains supplementary material available at 10.1186/s12862-024-02248-9.

## Introduction

Symbiotic bacteria play an important role in the life activities of many organisms, including insects and animals [1]. These bacteria reside in the host’s intestinal tract, epidermis and various organs, and jointly regulate each other’s life activities. The number of symbiotic bacteria even far exceeds the number of cells of the organism itself, and the diversity and the functions of symbiotic bacteria are vast, ranging from digestion and absorption [2–4] to detoxification, metabolism [5, 6], controlling the growth and reproduction of the host [7, 8], and adaptation to the environment [9–11].

Karst caves are a unique environment characterized by low air mobility, long-term darkness, high humidity, malnutrition, and isolation from the external environment. Researchers have studied microbial communities in small habitats such as cave rock walls [12], sediments [13], water and air [14]. The dominant phyla in these communities were Proteobacteria, Actinobacteria, and Firmicutes, although their relative abundance varied greatly among different caves [14]. Cave-dwelling organisms are also isolated from the outside world for a long time, which leads to the development of unique structures and habits. As a result, the cave-dwelling creatures differ from their counterparts outside the cave, both in their living environment and genetic relationships [15, 16]. In recent years, there have been a few studies on cave symbiotic bacteria. One study compared the symbiotic bacteria of *Oculina patagonica* obtained from bleached, cave and healthy corals and found a significant change in bacterial population in the absence of light [17]. Another study investigated cave calcium bodies in terrestrial isopod crustaceans *Titanethes albus* and *Hyloniscus riparius* [18].

Most studies on symbiotic bacteria of arachnids focus on the effects of endosymbiont on spiders [19, 20], mites [21], pseudoscorpions [22, 23] and other arachnids. However, relatively few studies have examined the entire bacterial community. One study of *Marpiss magister* found that endosymbionts are not the only microflora in spiders, and there are other bacterial groups in their bacterial communities [24]. Opiliones (harvestmen, shepherd spiders, daddy longlegs, etc.) are one of the most abundant and the oldest Arachnida. They are often widely distributed in ecosystems, independent *Cardinium* groups have been found in them. Some scholars use Opiliones as a control while studying *Wolbachia* and *Cardinium* infection of Araneae [25–28]. So far, studies on the symbiotic bacteria of Opiliones have mainly focused on single endosymbiont. To the best of our knowledge, no reports have been found on Opiliones-related bacteria based on different habitats and their effects on the growth, evolution and reproduction of the host. Opiliones have become an ideal subject for studying biological habitats due to their limited activity and narrow distribution areas [29, 30].

In this study, high-throughput technology (16S ribosomal RNA [rRNA] gene sequencing method) was used to analyze the relative abundance and diversity of symbiotic bacteria in different habitats of Opiliones and other arachnids, provide a basis for revealing the special material cycle in the cave environment, especially the nitrogen cycle. A variety of symbiotic bacteria have reproductive regulation and even co-evolution on the host. The comparison of symbiotic bacteria in Opiliones from different karst caves and habitats provided insights into the role of bacteria in the development and evolution of Opiliones and their distribution of the host.

## Materials and methods

### Sample collection

**Fig. 1:**
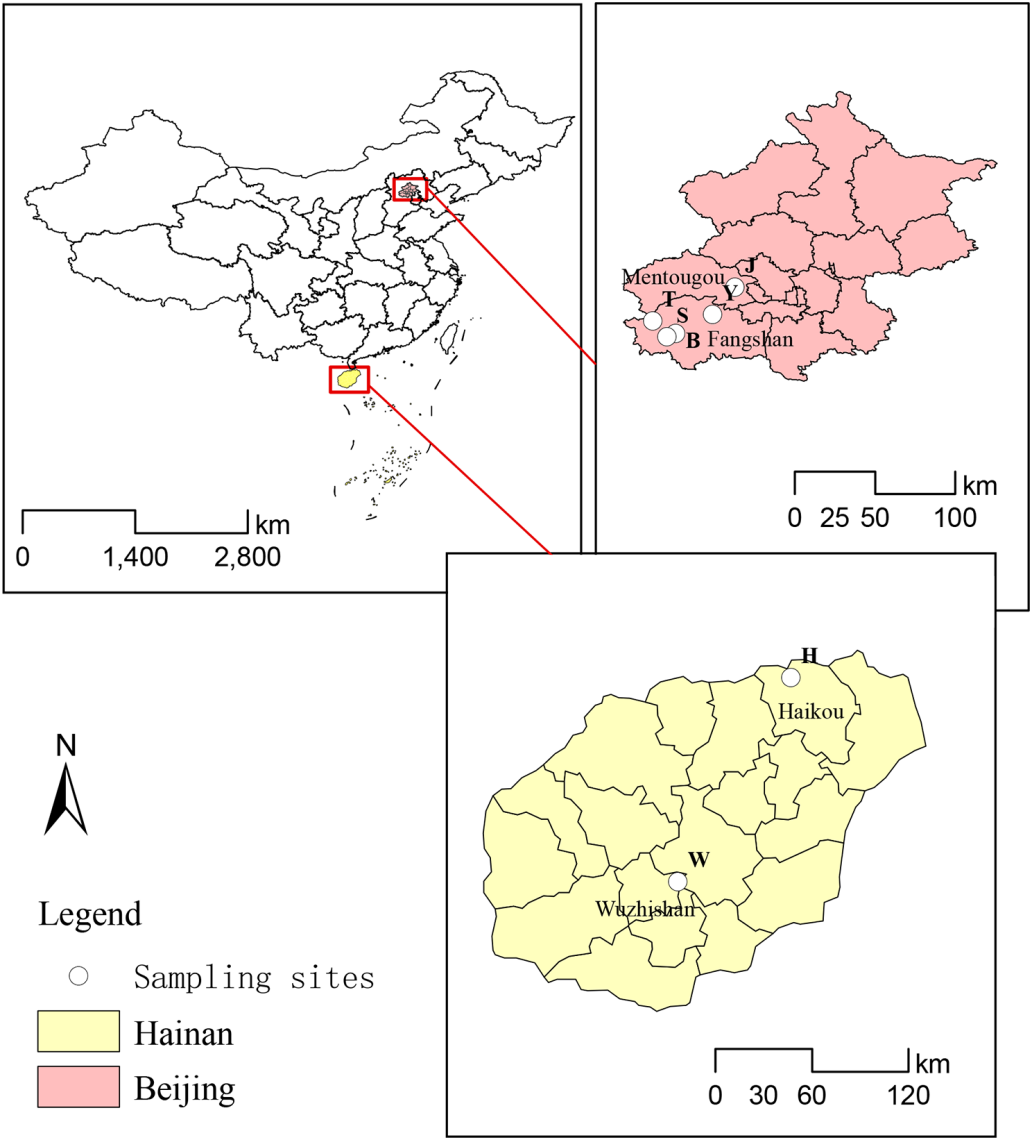
Sampling sites in Beijing and Hainan Island. The location number corresponds to the first letters of the sample number

**Fig. 2:**
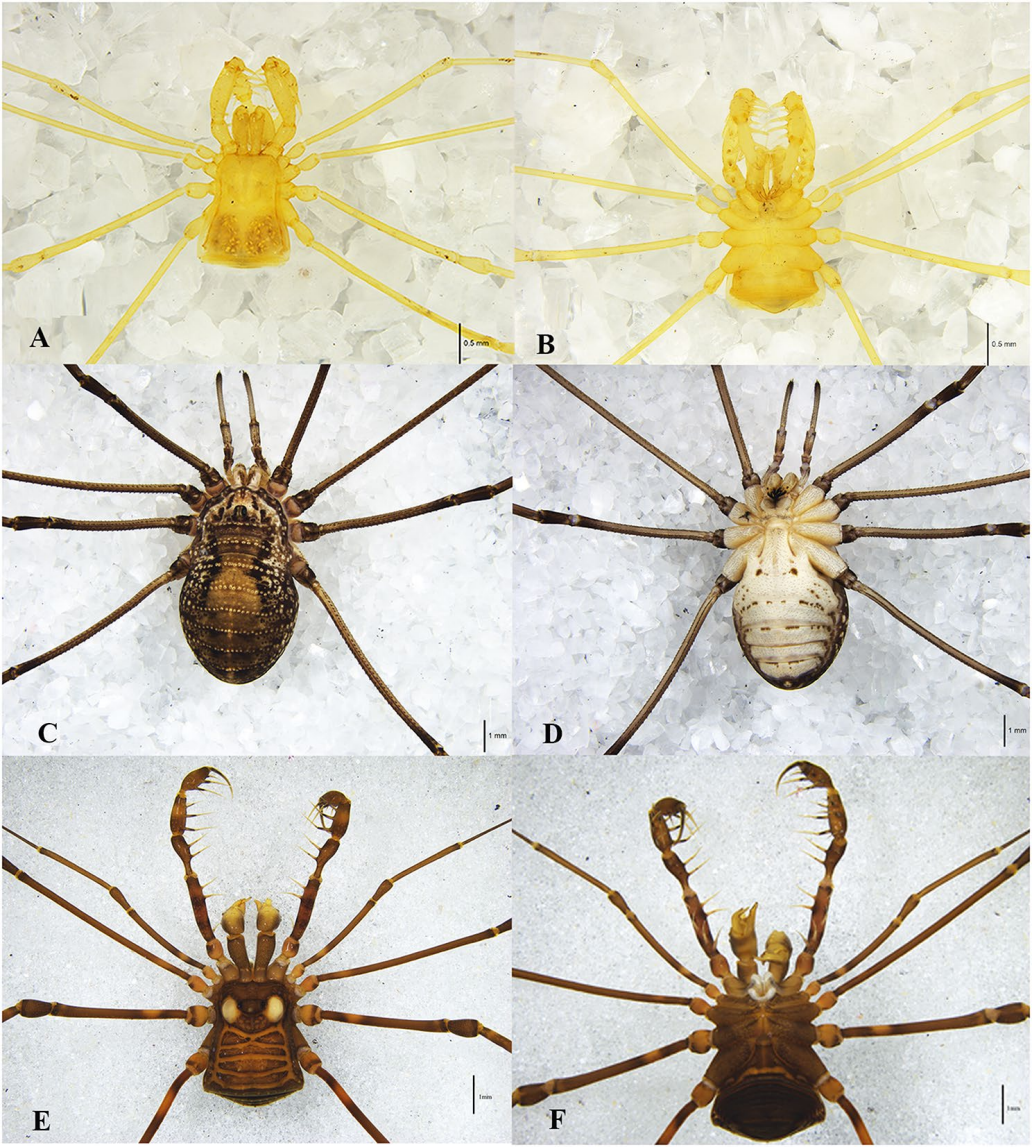
Photographs of some Opiliones. (**A, B**) *Sinonychia martensi*, Laniatores; (**C, D**) *Homolophus serrulatus*, Eupnoi; (**E, F**) *Euepedanus flavimaculatus*, Laniatores. (**A, C, E**) dorsal view, (**B, D, F**) ventral view

During the period from October 2021 to July 2022, five species of Opiliones were collected in Beijing and Hainan Island: *Sinonychia martensi* (Arachnida: Opiliones: Laniatores), *Euepedanus flavimaculatus* (Arachnida: Opiliones: Laniatores), *Plistobunus columnarius* (Arachnida: Opiliones: Laniatores), *Himalphalangium palpalis* (Arachnida: Opiliones: Eupnoi), *Homolophus serrulatus* (Arachnida: Opiliones: Eupnoi). Additionally, other Arachnida, including *Pimoa clavata* (Arachnida: Araneae), *Spelaeochthonius* sp. (Arachnida: Pseudoscorpionida) and *Draconarius* sp. (Arachnida: Araneae) were collected from various sections of Siyu Cave in Fangshan District (Fig. 1; Table 1). All samples were identified using traditional morphological classification (Fig. 2) and preserved in 95% ethanol.

**Table 1:** Samples were collected from different areas and caves

Family	Genus	Species	Site	Latitude and longitude	Habitat	Group
Epedanidae	*Euepedanus*	*E. flavimaculatus*	Haikou City, Hainan Province	20°0′43.44″N, 110°18′56.57″E	Forest, bush	HOF
	*Plistobunus*	*P. columnarius*	Wuzhishan City, Hainan Province	18°52′8.54″N, 109°40′57.71″E	Forest, bush	WOC
Cladonychiidae	*Sinonychia*	*S. martensi*	Bianfu Cave, Beijing (Fangshan)	39°42’23.94"N, 115°43’04.63"E	The deep zone of the cave	BOM
			Siyu Cave, Beijing (Fangshan)	39°41′1689″N, 115°40′4.83″E	The deep zone of the cave	SOM
			You Cave Beijing (Fangshan)	39°48′47.62″N, 115°55′21.19″E	The deep zone of the cave	YOM
Sclerosomatidae	*Himalphalangium*	*H. palpalis*	Tangshang Village, Beijing (Fangshan)	39°46′42.71″N, 115°35′20.57″E	Forest, bush	TOP
	*Homolophus*	*H. serrulatus*	Jingxi Ancient Road, Beijing (Mentougou)	39°58′0.573″N, 116°2′59.07″E	Forest, bush	JOS
			Tangshang Village, Beijing (Fangshan)	39°46′42.71″N, 115°35′20.57″E	Forest, bush	TOS
Pseudotyrannochthoniidae	*Spelaeochthonius*	*Spelaeochthonius* sp.	Siyu Cave, Beijing (Fangshan)	39°41′1689″N, 115°40′4.8252″E	The deep zone of the cave	SPS
Pimoidae	*Pimoa*	*P. clavata*	Siyu Cave, Beijing (Fangshan)	39°41′1689″N, 115°40′4.8252″E	The deep zone of the cave	SAC
Agelenidae	*Draconarius*	*Draconarius* sp.	Siyu Cave, Beijing (Fangshan)	39°41′1689″N, 115°40′4.8252″E	Within 5 m of the entrance	SAS

*Note* The deep zone of the cave: completely dark, high humidity and constant temperature. With the exception of *S. martensi, P. clavata* and *Spelaeochthonius* sp., other species were collected outside the cave. The three letters of the group name represent the site, order and species, respectively. For example, SOM corresponds to the Siyu Cave, Opiliones and *martensi*

### Sample processing and DNA extraction

The collected samples were thoroughly rinsed with sterile water 2–3 times, followed by sequential sterilization using 75% ethanol and hypochlorite, and finally washed again with sterile water. The processed samples were then stored at a temperature of -80℃ until DNA extraction. The extracted DNA was quantified using Nanodrop, and the quality of the extracted DNA was verified by running it through 1.2% agarose gel electrophoresis. The obtained DNA was stored at -20℃ for future use.

### Amplicon and library preparation

The 16S rRNA gene library construction and sequencing were performed by Personalbio Technology Company (Shanghai, China). The V3-V4 regions of the 16S rRNA gene were amplified using the primer pair 338 F/806R (5′-ACTCCTACGGGAGGCAGCA-3′ and 5′-GGACTACHVGGGTWTCTAAT-3′). PCR products were quantified using the PicoGreen dsDNA Assay Kit (Invitrogen, Carlsbad, USA), and samples were mixed according to the corresponding proportion based on the fluorescence quantitative results. The sequencing library was prepared using the TruSeq Nano DNA LT Library Prep Kit of the Illumina platform (Illumina, USA), and before sequencing, the library was checked. The qualified sequencing libraries were diluted, mixed, and denatured for computer sequencing.

### Data processing and analysis

The original sequences were quality screened and divided into libraries and samples according to index and Barcode information, and the barcode sequences were removed. The primer fragments were removed using qiime cutadapt trim-paired sequence, and unmatched primers were discarded. The qiime dada2 denoise-paired was used for quality control, denoising, splicing and de-chimerism. After denoising, all libraries, the ASVs feature sequence and the ASV table were merged, and singletons ASVs (ASV with a total sequence of only 1 in all samples) were removed [31, 32]. Annotation information was obtained using different algorithms and parameters in different databases, including the Silva database (http://www.arb-silva.de), nt database (http://ftp://ftp.ncbi.nih.gov/blast/db/), nr database (http://ftp://ftp.ncbi.nih.gov/blast/db/), based on the feature sequence of ASVs [33].

The composition and relative abundance of bacterial groups in each sample at different taxonomic levels were obtained by QIIME2 software (2019.4) [34]. The richness and diversity of bacteria in different samples were analyzed using alpha diversity indices (including Chao1 richness estimator, Observed_species, Shannon diversity index, and Simpson index) [35]. Beta diversity analysis was performed to analyze differences in bacterial community composition structure among different samples, and beta diversity was evaluated by PCoA and Non-metric multidimensional scaling (NMDS) based on the weighted Bray-Curtis distance algorithm [35, 36]. According to the abundance data of ASVs in all samples, the Wenn diagram was made according to the grouping of samples and calculating the relationship between each set. Indicator groups in different habitats were obtained by the LEfSe (the least discriminant analysis effect size) method with the LDA (linear discriminant analysis) threshold set to 4.0 [37]. The similarity between samples was shown in the form of hierarchical tree, and the clustering effect was measured by the branch length of the clustering tree. The heat map was used to analyze the species composition and displayed the species abundance distribution trend of each sample.

PICRUSt2 (Phylogenetic Investigation of Communities by Reconstruction of Unobserved States) was utilized to predict the functional abundance of samples based on the sequence abundance of marker genes in these samples [38]. FAPROTAX (Functional Annotation of Prokaryotic Taxa) is a functional annotation database specifically designed for prokaryotic taxa, enabling the functional prediction of bacterial communities [39]. It can be employed to evaluate bacterial metabolism and other ecologically related functions in cave environments, particularly focusing on the cycling function of sulfur, carbon, hydrogen and nitrogen.

## Results

### Diversity of the bacteria in Opiliones among different karst caves

**Fig. 3:**
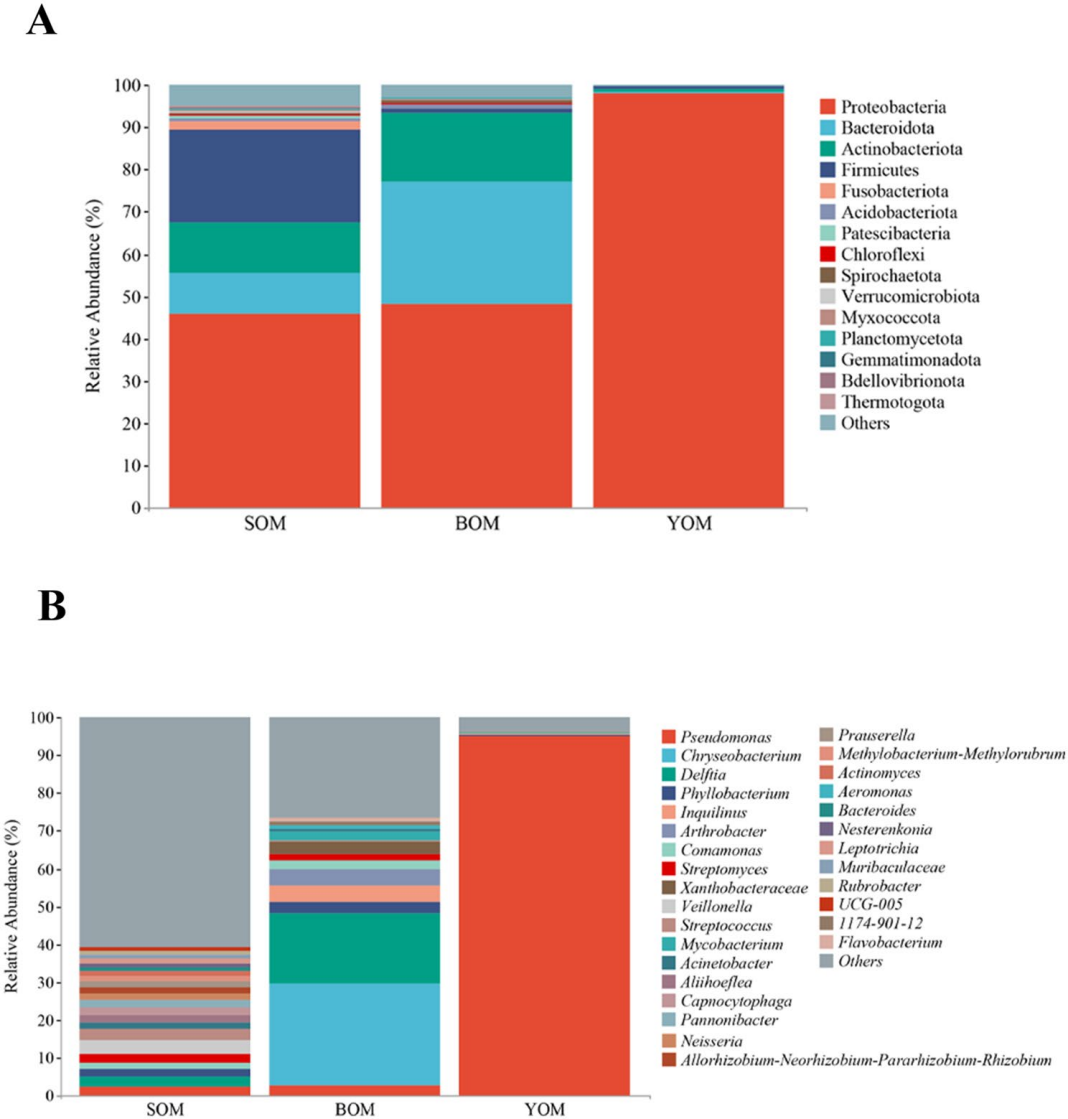
Relative abundance of *S. martensi* in Siyu, Bianfu and You Cave. (**A**) The relative abundance of phylum level in *S. martensi*. It shows the top 15. (**B**) Relative abundance of genus level in *S. martensi*. It shows the top 30. The relative abundance of other species merged and classified as Others

A study was conducted to investigate the diversity of bacteria in *Sinonychia martensi*, a species of harvestmen found in karst caves in Beijing. The results revealed significant differences in community structure and relative abundance of bacteria among different caves. In *S. martensi* inhabiting caves, the dominant phylum was Proteobacteria, with a much higher relative abundance compared to other bacteria. Proteobacteria accounted for an extremely high relative abundance of 98.06% in the samples from the You Cave (YOM) (Fig. 3a, Table S1). The relative abundance of Firmicutes in the Siyu Cave was 21.90%, second only to Proteobacteria, compared to the Bianfu Cave and You Cave. The three most abundance phyla accounted for more than 93% of the total microflora of *S. martensi* from the Bianfu Cave (BOM): Proteobacteria (48.10%), Bacteroidetes (29.01%), Actinobacteria (16.22%). At the genus level, *Pseudomonas* was the dominant bacteria in *S. martensi* from the You Cave (YOM), with a relative abundance as high as 95.17% (Fig. 3b, Table S1). The relative abundance of *Chryseobacterium* and *Delftia* in the samples from the Bianfu Cave (BOM) was 27.00% and 18.37%, respectively.

**Fig. 4:**
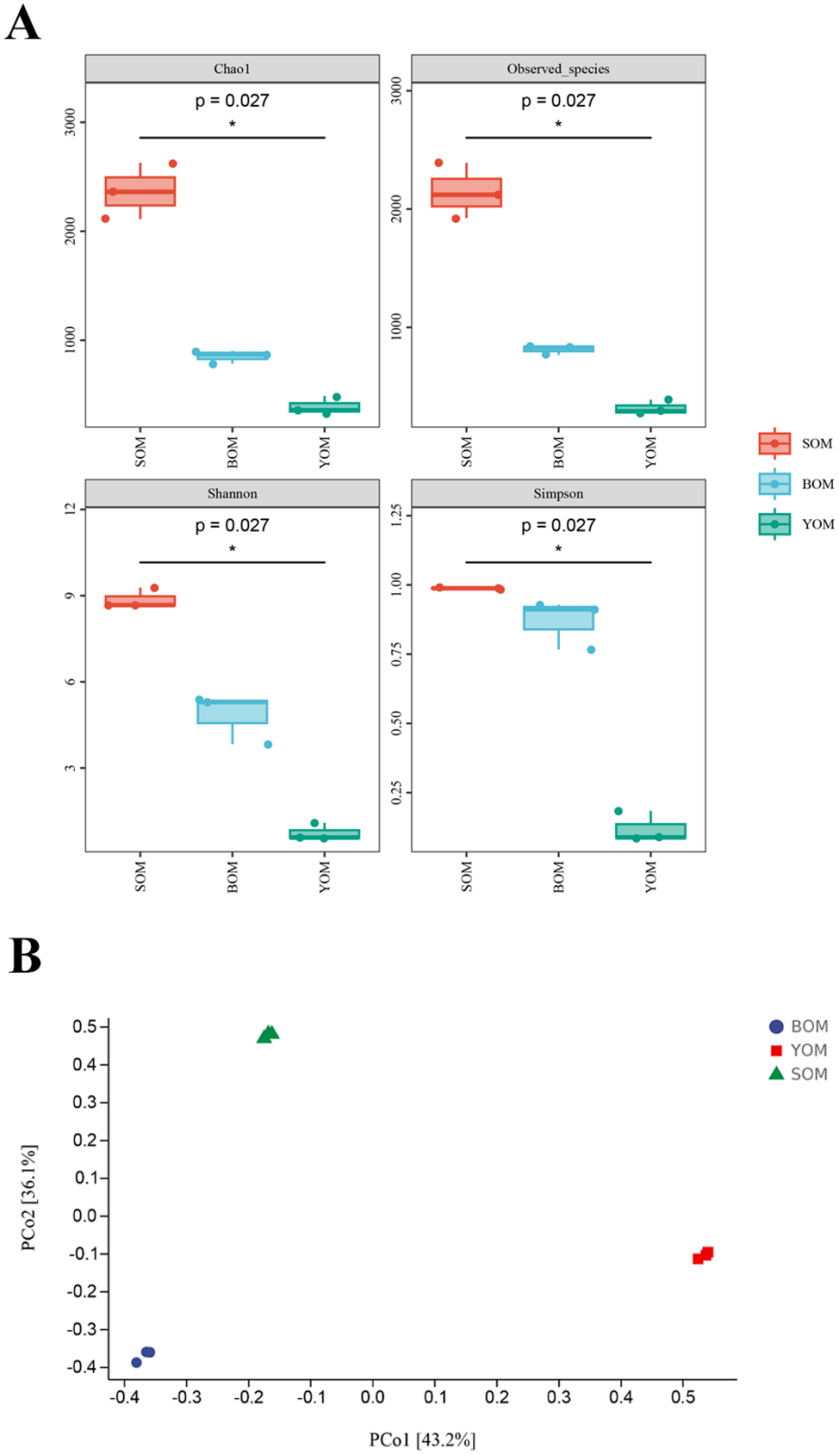
Comparison of microbial diversity in different cave samples. (**A**) Alpha-diversity analysis of *S. martensi* microbiome. Species richness and diversity were measured by Shannon, Simpson, Chao1 and Observed_species. Each panel corresponds to an alpha-diversity index, which is identified in the gray area at its top. In each panel, the abscissa is the grouping label, and the ordinate is the value of the corresponding alpha diversity index. The number under the diversity index label is the P value of the Kruskal-Wallis test. (**B**) Principal coordinate analysis (PCoA) of microbiome based on Bray-Curtis distances

The alpha diversity of bacteria in different caves was assessed by Chao 1, Observed_species, Shannon and Simpson indices (Fig. 4a). A Kruskal–Wallis test revealed a significant difference in alpha diversities among the caves (*P* ≤ 0.05). According to the Dunn’s post hoc test, the diversity and richness of *S. martensi* from the Siyu Cave (SOM) were significantly higher than those from You Cave (YOM) (*P* < 0.05). The beta diversity was evaluated by principal coordinate analysis (PCoA) based on the weighted Bray-Curtisdistance algorithm, showing that Opiliones samples were loosely clustered (Fig. 4b). In the first dimension, the community composition of the Opiliones samples from Siyu Cave (SOM) was similar to that of the Bianfu Cave (BOM). It was more similar for symbiotic bacteria from You Cave (YOM) and Bianfu Cave (BOM) in the second dimension. These findings suggest that caves, as unique and enclosed habitats, harbor distinct symbiotic bacteria across different caves

**Fig. 5:**
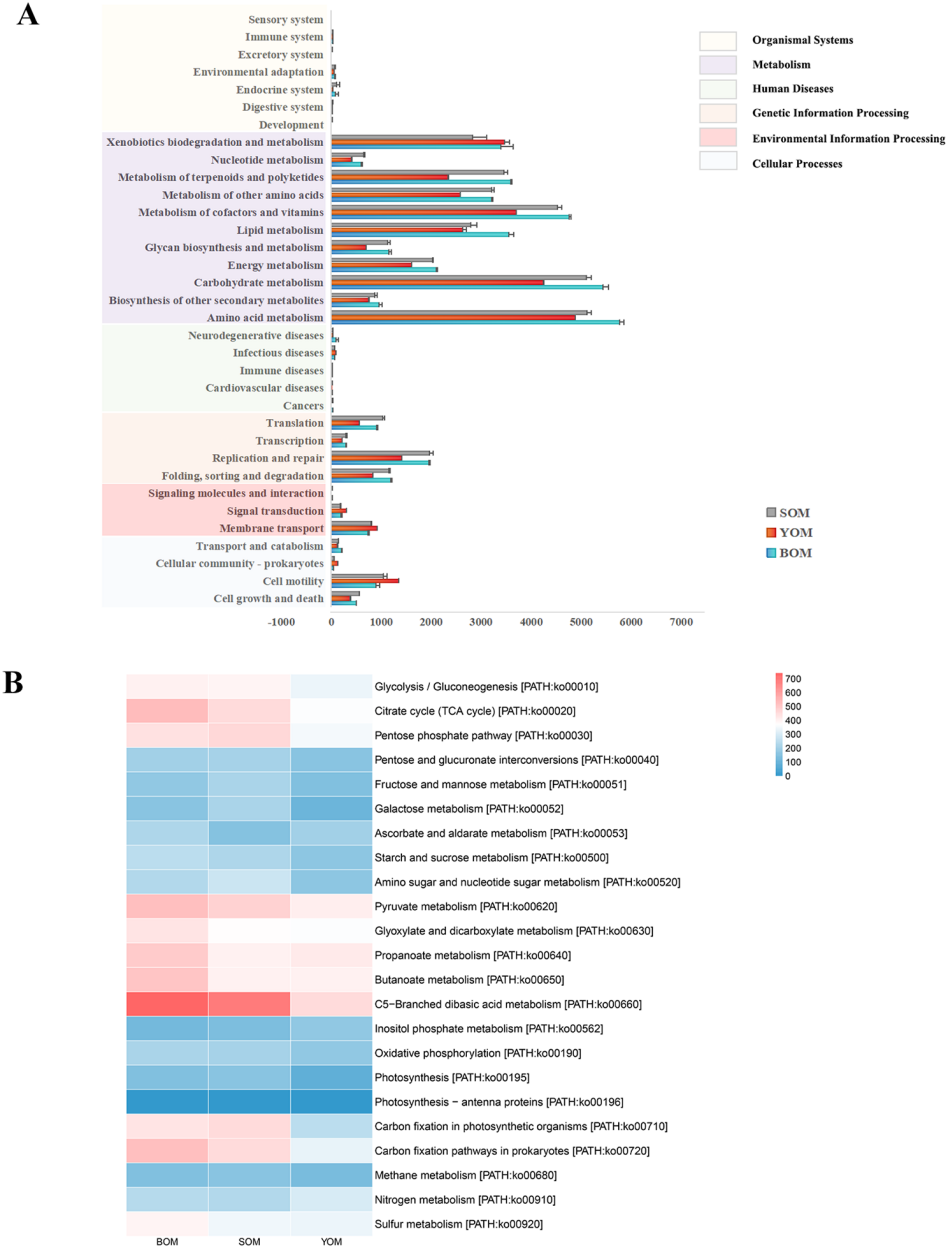
Predicted bacterial function in cave samples using PICRUSt2. (**A**) Predicted function of bacteria among the five samples. The first level of the KEGG pathway was represented by different colors. (**B**) The second level of the KEGG pathway was shown in the heatmap. It included only carbohydrate metabolism and energy metabolism pathways

**Fig. 6:**
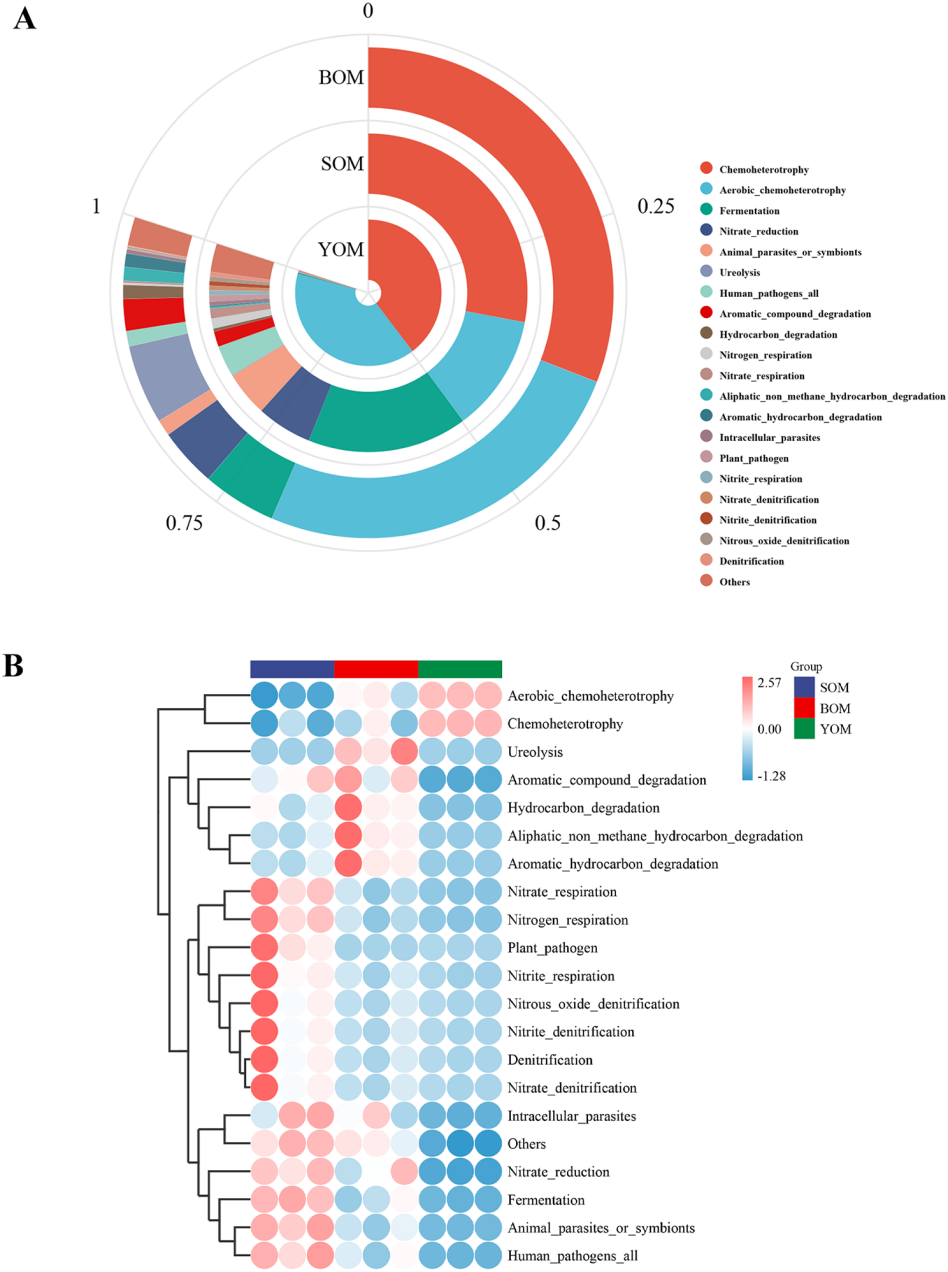
Predicted bacterial function in cave samples using FAPROTAX. It showed the top 20 functions predicted. (**A**) Abundance of the main metabolisms in the whole set of cave samples. (**B**) Heatmap of the FAPROTAX analysis revealed the differences among samples

Caves represent unique habitats with distinct energy flows and material circulations compared to the outside environment, and each creature has adapted unique behaviors to survive. To understand the function of bacteria in different caves, PICRUSt2 software [18] was used to predict and analyze the symbionts. The KEGG (Kyoto Encyclopedia of Genes and Genomes) database was used to identify six functional modules, including cellular processes, environmental information processing, genetic information processing, human diseases, metabolism, and organismal systems. The metabolism module was found to be the most abundant functional module in all samples (Fig. 5a), which might include the glycolysis pathway, pentose phosphate pathway, nitrogen metabolism, sulfur metabolism, methane metabolism and multiple carbon sequestration pathways (Fig. 5b). The relative abundance of pathways was in different caves and samples. The COG (clusters of orthologs groups) database was used to predict key enzymes in multiple reaction pathways (Table S2), including Hexokinase [EC:2.7.1.1], glyceraldehyde 3-phosphate dehydrogenase [EC:1.2.1.12] and pyruvate kinase [EC:2.7.1.40] involved in the glycolysis pathway. Glucose-6-phosphate isomerase [EC:5.3.1.9] is involved in the pentose phosphate pathway. Nitrite reductase [EC1.7.2.1] catalyzes the reduction of nitrite (NIT), a crucial enzyme in the nitrogen cycle in nature. Chemoheterotrophy and aerobic chemoheterotrophy were found to be the most abundant functions based on the predictions made by FAPROTAX analyses (Fig. 6a). Among the top 20 predicted functions, nine were related to the nitrogen cycle, including nitrate reduction, nitrogen respiration, nitrate respiration, ureolysis, nitrification, nitrite respiration, nitrate denitrification, nitrite denitrification and nitrous oxide denitrification. Specifically, the SOM samples showed high abundance in the functions related to the nitrogen cycle and the BOM samples were positively correlated with the degradation function of organic compounds (Fig. 6b). These findings indicate that different caves employ different strategies in the nutrient cycle

### Differences in the symbiotic bacteria between Opiliones inside and outside the cave

**Fig. 7:**
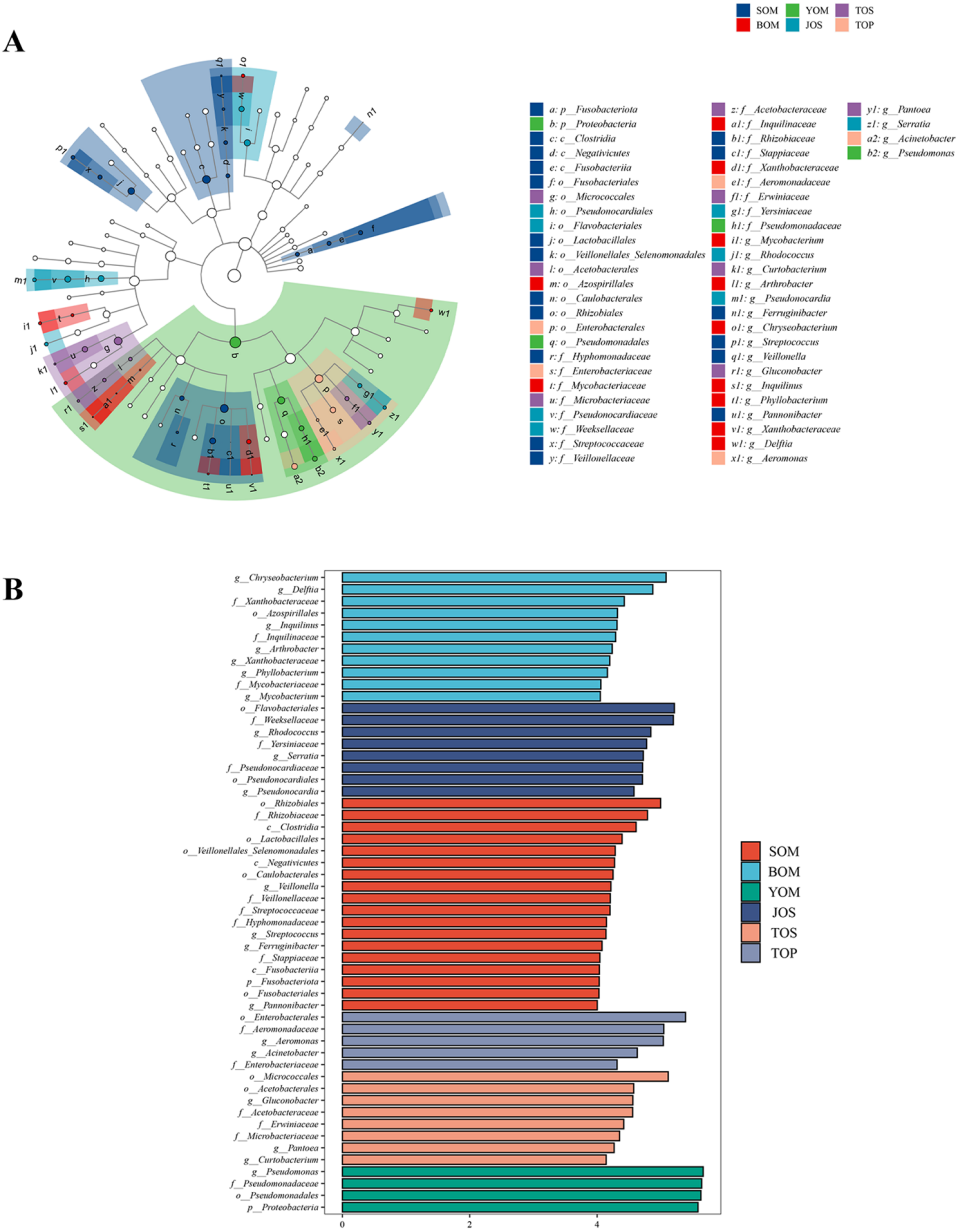
Results of LEfSe analysis. (**A**) Cladograms indicating the phylogenetic distribution of bacterial lineages associated with Opiliones inside and outside the cave. (**B**) Indicator bacterial group significantly differentiated across the three sample types with LDA values was 4

**Fig. 8:**
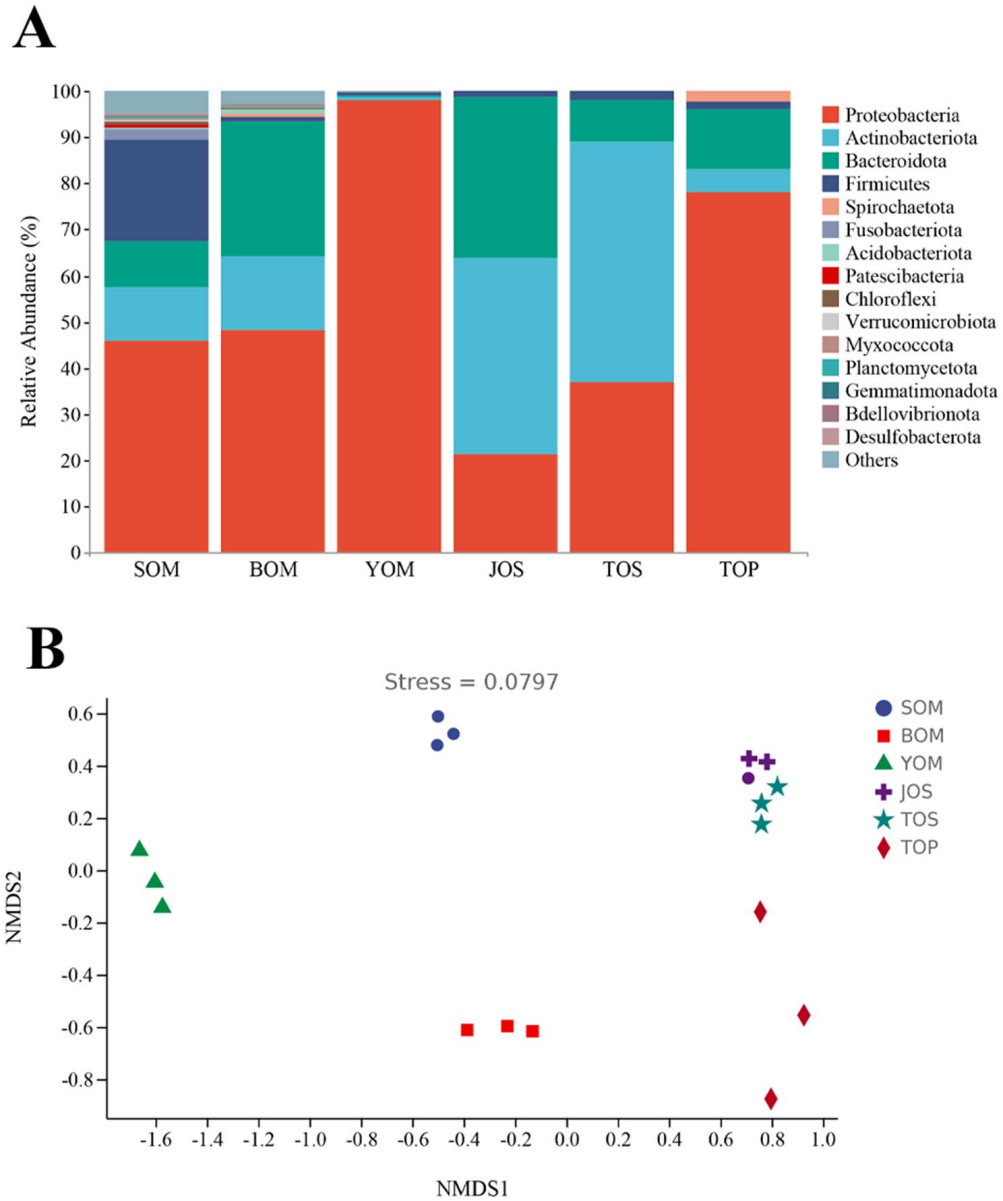
Diversity of symbiotic bacteria from Opiliones inside and outside caves in Beijing. (**A**) Relative abundance of Opiliones inside and outside caves. It showed the top 15 phyla and the relative abundance of other species merged and classified as Others. (**B**) Non-metric multidimensional scaling (NMDS) plots of microbiome difference at ASV level based on Bray-Curtis distances. The stress was 0.0797

The sequencing results of *Himalphalangium palpalis* and *Homolophus serrulatus* outside the caves and *S. martensi* inside the cave revealed differences in the bacteria structure of symbiotic bacteria inside and outside the cave were different. LEfSe analysis was performed to compare bacterial communities to find the specialized bacterial groups within each type of the sample (Fig. 7a). The cladogram showed that 2 phyla, 3 classes, 12 orders, 17 families, and 20 genera were significantly variable across the five samples. When the LDA value was set to 4, the number of differentially abundant bacterial groups in the five samples were 11 (BOM), 8 (JOS),18 (SOM), 5 (TOP), 8 (TOS)and 4 (YOM), respectively (Fig. 7b). At the phylum level, Proteobacteria was the main dominant group in cave Opiliones, accounting for 98.06%, 48.10% and 45.80% in the three caves, respectively (Fig. 8a, Table S3). In contrast, Actinobacteria and Proteobacteria were differentially distributed in samples outside the cave, with Actinobacteria having the highest relative abundance in *Homolophus serrulatus* (TOS and JOS), and Proteobacteria being dominant in *Himalphalangium palpalis* (TOP). The NMDS plots based on Bray-Curtis distances clearly distinguished the Opiliones inside the cave from those outside the cave (Fig. 8b).

### Effect of cave environment on symbiotic bacteria of Arachnida

**Fig. 9:**
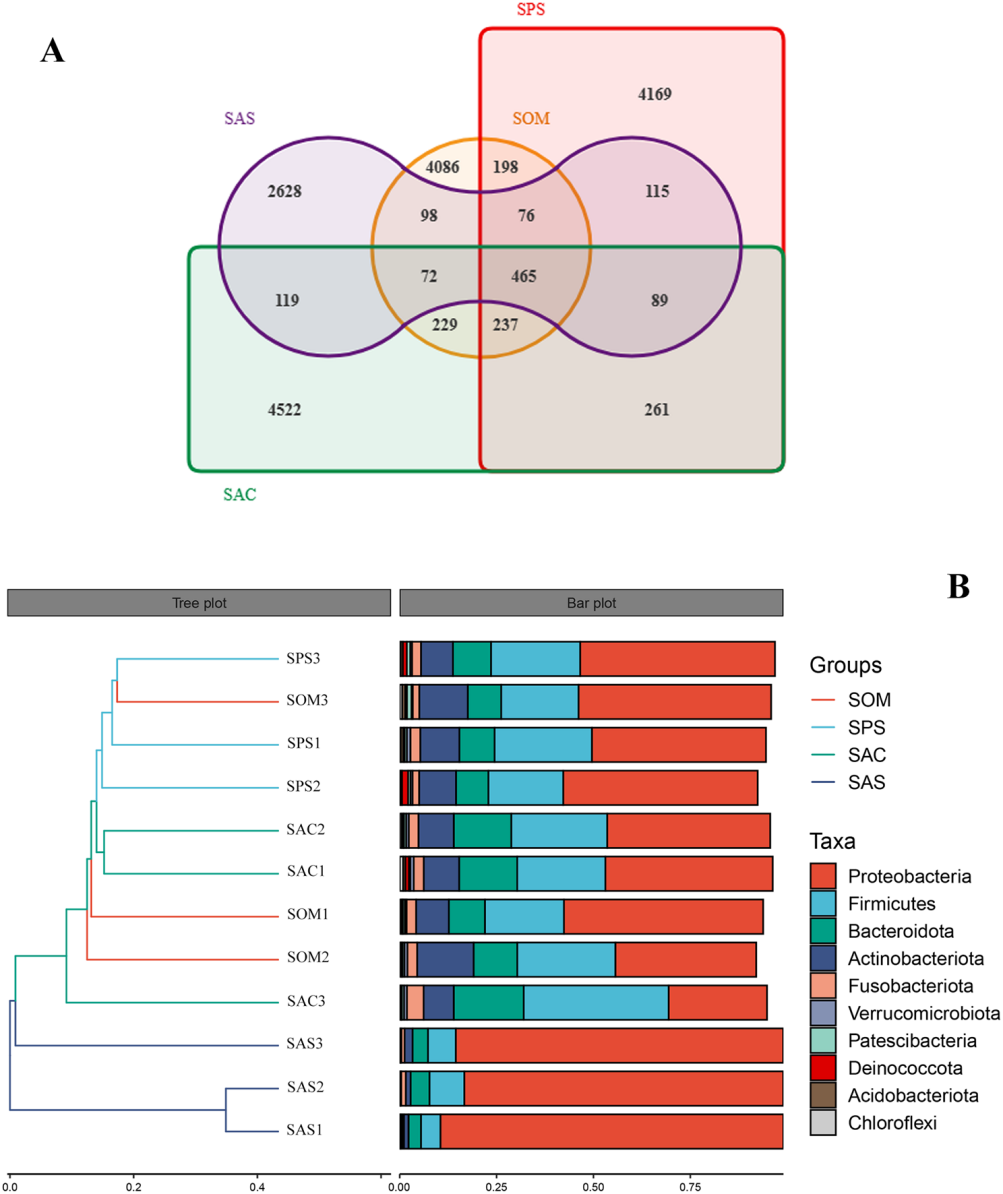
Comparative microbial diversity of Arachnida in Siyu Cave using bate-diversity analysis. (**A**) Venn diagram of Siyu Cave samples. (**B**) Hierarchical clustering analysis of the similarity between samples of Siyu Cave. The hierarchical clustering analysis was utilized to demonstrate the similarity between the samples, represented by a hierarchical tree in the left panel. The samples were clustered based on their similarity, with shorter branch lengths indicating greater similarity. The right panel shows the top 10 phyla in a stacked histogram

A study was conducted to investigate the effect of the cave environment on the symbiotic bacteria of three different Arachnida species: *S. martensi* (Opiliones), *P. clavata* (Araneae) and *Spelaeochthonius* sp. (Pseudoscorpionida) inside the Siyu Cave. The results showed that the bacteria structure in the samples was similar. Proteobacteria, Firmicutes, Bacteroidetes and Actinobacteria were consistently prevalent phyla among all species inside the cave, accounting for a comparable proportion (Figure S1a, Table S4). *Draconarius* sp. (Arachnida: Araneae) collected outside the cave differs from that inside. The relative abundance of Proteobacteria in the SAS was 85.10%, which was significantly higher than that inside the cave. At the genus level, the relative abundance of *Wolbachia* and *Rickettsia* in SAS was 49.52% and 23.82% respectively, whereas other bacteria were less than 1.5% (Figure S1b, Table S4). The diversity and richness of the symbiotic bacteria inside the cave were higher than those outside the cave (Figure S1c). The Venn diagram revealed that there were differences in ASVs among the four samples. A total of 465 ASVs were shared among all four samples and the cave samples shared 237 ASVs. The number of ASVs unique to spiders outside the Siyu Cave (SAS) was 2628 (Fig. 9a). The members of SAS were loosely clustered, which was confirmed in the hierarchical clustering diagram based on Bray-Curtis distance level and histograms of species composition (Fig. 9b).

### Some similarities in environment between karst caves and Hainan Island

We conducted a comparison of symbiotic diversity between Opiliones from Hainan Island and those inside Beijing caves. To reflect their similarity, we utilized Opiliones outside the Beijing caves as a control group. At the phylum level, Proteobacteria was the dominant bacteria across all samples, with relative abundances of 98.06%, 92.36% and 78.18% in YOM, HOF and TOP, respectively (Figure S2a, Table S5). However, Actinobacteria was the main dominant bacteria in JOS and TOS, with lower levels in other classifications. Firmicutes emerged as the secondary dominant bacteria in HOF, WOC and SOM, with the relative abundance of 6.31%, 20.17%, and 21.90% respectively.

The taxonomic alpha-diversities measured by Simpson indicated significant differences in the diversity of symbiotic bacteria among different groups (*P* < 0.05). Furthermore, Shannon, Chao1 and Observed_species indicated significant differences in diversity and richness (*P* ≤ 0.01). Notably, Chao1 and Observed_species indicated differences between HOF and SOM, and significant diversity differences were observed between YOM and TOS (Figure S2b).

**Fig. 10:**
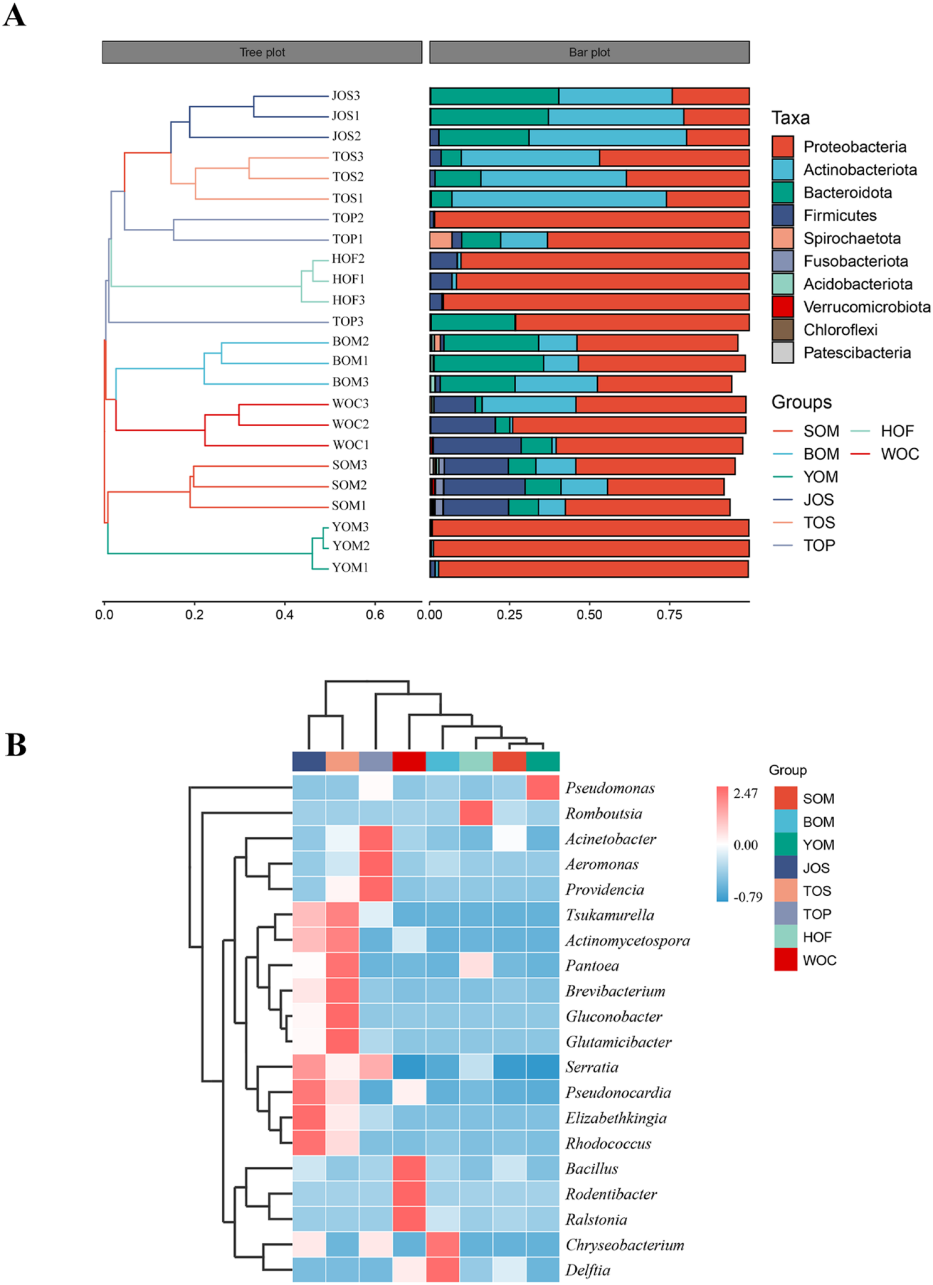
Comparative microbial diversity of Opiliones in Beijing caves and Hainan using bate-diversity analysis. (**A**) Hierarchical clustering analysis of the similarity between samples in the form of the hierarchical tree. (**B**) Heatmap of the microbial composition and relative abundance of Opiliones

The population structure and alpha-diversity analyses revealed similarities between Opiliones from Hainan Island and those inhabiting the Beijing cave. Therefore, beta analysis methods, indicated distinct symbiont structures among Opiliones living in different regions, with symbiotic bacteria in Opiliones from Hainan Island and the Beijing caves showing greater similarities. Cluster analysis based on the Bray-Curtis distance level grouped BOM and WOC together (Fig. 10a). Similarly, JOS, TOP and TOS were clustered into a group, except that TOP3 was independent of them. Additionally, WOC, BOM, HOF, YOM and SOM clustered together in the heat map of UPGMA clustering based on the Euclidean distance of species composition data (Fig. 10b). Specifically, WOC and BOM were positively correlated with *Bacillus*, *Rodentibacter*, *Ralstonia* and *Chryseobacterium*, *Delftia* respectively, and clustered together. *Pseudomonas*, which belonged to YOM, also clustered with the aforementioned groups. The results confirmed that the symbiotic bacteria of Opiliones from Hainan Island and Beijing caves were more similar than those outside the cave.

## Discussion

Karst caves are unique habitats that are characterized by specific environmental conditions, such as almost constant temperature and humidity, permanent darkness and limited energy and nutrient sources [40]. These conditions exert a profound influence on the diversity and composition of cave ecosystems [41]. In addition, caves are mostly located in remote areas and are less disturbed by human activities, which makes them ideal locations for studying the underground biosphere. However, the cave environment is limited by many factors, such as restricted access to nutrition, low temperatures, unique air pressure conditions, connectivity with groundwater, variations in light and humidity, limited surface interaction, anthropogenic or animal influences and geological mineral compositions [42–44]. So each cave is unique in its biological, chemical and physical characteristics, and these factors cause differences within and between caves [45]. These disparities elucidate the variations in symbiotic diversity observed among Opiliones inhabiting different caves and those residing outside cave systems in this study.

Karst caves are relatively closed environments, there are obvious differences among different caves. The cave habitat serves as a critical factor influencing the survival and community structure of microorganisms, with these organisms occupying diverse niches in space and time. It is worth noting that each cave has a unique microbial community, which emphasizes the important role of the cave niche in shaping microbial diversity [46]. Moreover, the cave environment exerts a strong impact on the organisms inhabiting the cave, with up to five distinct zones identified in caves, including the entrance, twilight, transition, deep, and stagnant air zones, each harboring a distinct biological community. The transition zone, characterized by complete darkness and variable abiotic environment [16], and the deep zone, completely dark with high humidity and constant temperature, are of particular interest. *S. martensi* (Fig. 2a, b) observed in this study is found, which is adapted to the completely lightless deep zone of the cave, displays body coloration varying from yellowish white to pale yellow, greatly reduced ocular size, and complete absence of eyes and retinae, indicating long-term adaptation to the cave environment [47]. Although the Opiliones from Siyu Cave, Bianfu Cave and You Cave share the same appearance and molecule, their different environments lead to distinct symbiotic bacteria structures (Fig. 3a, b). Furthermore, significant differences were observed in symbiotic diversity among Opiliones from the three caves (Fig. 4a, b).

The unique environment of karst caves, characterized by darkness and nutrient limitation, results in distinct energy flow and material circulation compared to the external environment. Predictive functional analysis using PICRUSt2 revealed that despite variations among different cave environments, symbiotic bacteria in Opiliones from three caves exhibit similar functions, focusing on metabolic activities such as the synthesis and metabolism of various organic compounds (Fig. 5a). Symbiotic bacteria rely on the host for various life activities, including even simple metabolic functions. The results suggest that *S. martensi* and its symbiotic bacteria play direct or indirect roles in the material cycle within the cave, particularly in the carbon cycle, which involves crucial metabolic processes like carbon fixation, methane metabolism, and carbon degradation. Annotation by KEGG and COG databases indicated that most symbiotic bacteria in Opiliones participate in diverse carbon fixation pathways, such as the glycolysis pathway, tricarboxylic acid cycle, and pentose phosphate pathway, facilitated by relevant enzymes (Fig. 5b, Table S2). Additionally, Opiliones are omnivores, with their diet primarily comprising invertebrates in caves, along with plants and fungi [48, 49]. Following the ingestion of animal and plant residues and other organic matter into the Opiliones’ intestinal tract, enzymatic decomposition within the digestive tract, coupled with interactions with intestinal symbiotic bacteria, facilitates the return of organic matter to the cave environment in the form of feces. This process enhances the utilization of other plants and microorganisms within the cave ecosystem.

Nitrogen is a vital element in organisms, playing a crucial role in many biochemical cycles within cave ecosystems. The symbiotic relationship between *S. martensi* and its associated bacteria contributes significantly to the nitrogen cycle within caves. Firstly, they actively participate in biological nitrogen fixation, a process where nitrogen-fixing bacteria convert atmospheric nitrogen into biologically available ammonia. The prevalence of Proteobacteria in the samples suggests their key role in the nitrogen cycle within the cave environment [50]. Several genera of Pseudomonadales, known for their association with nitrogen fixation, particularly *Pseudomonas*, are abundant in Opiliones samples from the You Cave (YOM) [51, 52]. Additionally, Actinobacteria, which are abundant in all samples, also contribute to the nitrogen cycle in various ecosystems [53, 54]. The presence of nitrogenase molybdenum-iron (MoFe) protein and other nitrogenases further supports biological nitrogen fixation (Table S2).

Secondly, *S. martensi* and its symbiotic bacteria facilitate denitrification, a critical aspect of the nitrogen cycle. Predictive analysis using FAPROTAX revealed that denitrification accounts for a significant proportion of nitrogen-related functions (Fig. 6a). Denitrification establishes a loop between atmospheric and ecological water bodies, with the process involving key enzymes such as nitrate reductase (Nar), nitrite reductase (Nir), nitric oxide reductase (Nor), and nitrous oxide reductase (Nos). These enzymes are present in the symbiotic bacteria of Opiliones from all three caves (Table S2). Aerobic denitrifying bacteria such as *Comamonas*, *Acinetobacter*, and *Pseudomonas*, known to facilitate denitrification, exhibit high abundance in Opiliones samples from various caves, particularly *Pseudomonas* [55, 56] (Fig. 3b).

The karst cave represents a terrestrial oligotrophic extreme underground ecosystem characterized by limited sources of total organic carbon and nitrogen. Within this environment, Opiliones and their symbiotic bacteria play integral roles in the unique energy and material cycles. Operating amidst constant darkness and low energy availability, they contribute significantly to the ecosystem’s functioning. These findings underscore the importance of investigating microbial communities within cave ecosystems to gain insights into their ecological processes and biogeochemical cycles. Understanding the dynamics of these symbiotic relationships and their impact on nutrient cycling sheds light on the intricate balance of life in these remote and often overlooked environments.

Many differences between the environment inside and outside the cave, and the community composition of symbiotic bacteria is also obviously different. The environment inside the cave remains relatively constant. This makes it worthwhile to study the unique community structure of symbiotic bacteria in comparison to the Opiliones living outside the cave, especially in the north where there are obvious changes in the four seasons. We conducted a comparison between *S. martensi* inside the cave and the *Himalphalangium palpalis* and *Homolophus serrulatus* outside the cave and found that there was a significant difference between them. At the phylum level, although most of the samples had Proteobacteria, Actinobacteria and Bacteroidetes as the dominant group, the abundance of symbiotic bacteria varied across different environmental samples (Fig. 8a). Comparing the Opiliones inside and outside of the cave, and a significant difference was found not only in the composition of the community but also in the species of dominant bacteria (Fig. 8a, b).

Two potential explanations may account for this phenomenon. First, the genetic relationship between *S. martensi* and *Himalphalangium palpalis*, *Homolophus serrulatus* is relatively distant. *S. martensi* belongs to the Laniatores group, characterized by robust tentacles (Fig. 2a, b, e, f) and a diet primarily consisting of other insects, while *Himalphalangium palpalis* and *Homolophus serrulatus* belong to the Eupnoi group, lacking strong tentacles (Fig. 2c, d) and capable of feeding on fungi, plants and animals. Previous studies have shown that genetic relationships can have a great influence on the structure of symbiotic bacteria in animals. In addition to the co-evolution of primary endosymbionts with the host [57, 58], there are also symbiotic bacteria that are vertically transmitted from parents and give priority to transmission to closely related hosts [19]. Genes can regulate the body structure of different species, creating distinct internal environments and resulting in different dominant phyla. Secondly, differences in cave environment compared to outside conditions also contribute to this disparity. The cave environment remains constant, rarely disturbed by external temperature, and the temperature is maintained at more than ten degrees Celsius all year round. Opiliones inhabiting caves are thus unaffected by cold winters and remain active throughout the year. In contrast, Beijing experiences distinct four seasons, with temperature dropping significantly below zero in winter. The breeding, feeding and activities of Opiliones living outside Beijing caves are greatly limited by climate change.

Based on the two points mentioned above, which has more influence? We collected different samples (harvestmen, spiders and pseudoscorpions) from Siyu Cave and compared them. According to the results, we found that Proteobacteria, Actinobacteria, Firmicutes, and Bacteroidetes were the main constituent phyla of the species in the cave, and they each accounted for a similar proportion (Figure S1a, b). Although they belong to different orders and have completely different feeding methods (spiders inject digestive juices into their prey, while harvestmen chew directly), there was no significant difference in the structure of symbiotic bacteria (Figure S1c).

Afterward, we studied the spiders outside the Siyu Cave (SAS). To reduce the impact of the external environment and geographical location, we collected samples near the entrance, where there is light and climate change, significantly differs from that in the cave. From inside the cave to the outside, there were obvious changes in the structure of microflora in spiders from different environments (Figure S1a, b). Compared with the spiders in the cave (SAC), the proportion of Proteobacteria in the symbiotic bacteria outside the cave (SAS) increased significantly at the phylum level, and at the genus level, *Wolbachia* and *Rickettsia* became the dominant flora. Both the diversity and richness of symbiotic bacteria in cave spiders were much higher than those outside the cave (Figure S1c). Although the spiders, harvestmen and pseudoscorpions in the same cave environment have obvious differences in genetic relationship, their symbiotic bacteria are highly similar. Compared with harvestmen and pseudoscorpions, spiders inside and outside the cave are significantly closer in genetic relationship and feeding methods, but there are significant differences in the community structure of symbiotic bacteria. Therefore, compared with heredity, the effect of the cave environment on symbiotic bacteria is more obvious.

Symbiotic bacteria are not fixed, and there are many factors affecting them, among which the environment plays an important role. Most of the microbes found in organisms are acquired from the surrounding environment during the growth process, either directly or indirectly. Significant seasonal fluctuations of microbial communities are observed in wild mice [59] and hibernating ground squirrels (*Ictidomys tridecemlineatus* and *Urocitellus parryii*) every year [60, 61]. Organisms residing in deep-sea hydrothermal regions characterized by high pressure, hypoxia, high levels of toxic substances, sulfides, and heavy metals, as well as being dark, are doubly symbiotic with two gammaproteobacterial endosymbionts: a sulfur oxidizer and a methane oxidizer [62, 63]. High temperatures can denature proteins, causing physiological and developmental problems [10]. Endosymbiosis with heat shock tolerance can reduce the impact of high temperature on the survival and development of the host [11]. Various factors such as season, altitude, diet and photoperiod impact the diversity of animal microbial communities [9]. The community composition of larvae of *Aedes aegypti*, *Aedes albopictus* and *Culex quinquefasciatus* from different areas varied greatly, while strong similarities were found among larvae of different species developing in the same site [64]. These findings, along with previous studies, suggest that the environment exerts a more significant influence on symbiotic bacteria than heredity. However, in this study, arachnids were the subjects, and their genetic relationships were relatively close. If we take more closely related animals from different phyla or classes as samples, it remains uncertain whether the environment would still play such a significant role. Furthermore, due to residing in the cave for an extended period, Araneae, Opiliones and Pseudoscorpionida have undergone convergent evolution to some extent, exhibiting similar morphological features, such as the lack of eyes and a light body color. Could this similarity also affect the symbiotic structure of these three groups, making them more alike? Further studies are necessary to verify this hypothesis.

It is indeed interesting that the symbiotic bacteria richness of cave organisms surpasses found that outside the cave despite the challenging environment. This suggests that the cave environment may provide a unique niche for symbiotic bacteria to thrive. The caves in this study are located in the north of the Taihang Mountains, within the center of the North China Craton (NCC). This area is known for its abundance of mineral deposits [65, 66]. *Phyllobacterium*, previously isolated from plant roots in mines, exhibits the capability to oxidize Mn (II) [67]. The abundance of *Phyllobacterium* in cave species (Table S5) may be due to its tolerance to heavy metals [68, 69], which are often present in mineral-rich cave environments. The joint action of nutrition, rock and other factors may contribute to the richness of symbiotic bacteria in caves [70, 71], and may also explain the observed difference in symbiotic bacteria between cave and non-cave organisms. Further studies are needed to fully understand the mechanisms underlying these observations.

*Sinonychia martensi* found in Beijing caves in this study is a highly adapted species to cave life, and the first superfamily Travunioidea discovered in China [72]. Currently, the Travunioidea are distributed across the Holarctic, in the temperate regions of central Europe, southern British Columbia, eastern and western United States, Japan, and South Korea [73]. Although *Sinonychia* is closely related to *Speleonychia* and shares some morphological similarities, it is considered a new genus. Interestingly, *Speleonychia*, closely related to *Sinonychia*, is mainly located in the Pacific Northwest of North America, across the Pacific Ocean from the *Sinonychia* [72]. The Laniatores, the order to which *Sinonychia* belongs, are mainly distributed in the tropical and temperate regions of the southern hemisphere [74] with more common occurrences in Hainan and Sichuan, situated in the south of China [75–77]. Therefore, it raises the question of why *S. martensi*, the only Travunioidea found in China, appears in caves in the north.

Two main hypotheses are generally proposed to explain the shift from surface to underground habitats. The first hypothesis suggests that the colonization of underground habitats is the result of the active expansion and colonization by a founder [78], rather than accidental stranding and persistence in lightless areas [79, 80]. The second hypothesis proposes that species may be forced to adapt to cave life due to environmental changes that make the surface uninhabitable [81–83]. The Opiliones in this study exhibit obvious characteristics of cave life, indicating that they have been forced to move underground and have lived at the bottom of the cave for many years. Therefore, *S. martensi*, being the first of its kind found in China, likely originated from the same southern family with a warm and humid climate rather than migrating all the way to the north.

Some similarities in symbiotic bacteria of Opiliones from Hainan Island and karst caves suggest that karst caves can serve as an alternative habitat for species thriving in warm and humid climates. However, does this imply that the cave environment is comparable to its natural habitat? Hainan Island, a typical tropical region in China, is home to a significant number of Laniatores species. In this study, *E. flavimaculatus* and *P. columnarius* were collected from Haikou City and Wuzhishan City of Hainan Island respectively, and compared with *S. martensi* from Beijing caves. To provide a clearer comparison, *Himalphalangium palpalis* and *Homolophus serrulatus*, also found outside the cave in Beijing, were included in the study. The results showed that the symbiotic structure of *S. martensi* was more similar to that of Opiliones from Hainan Island compared to Opiliones outside the cave in Beijing. The relative abundance of Proteobacteria in *Homolophus serrulatus* was only 21.44% and 37.07%, whereas it was the highest in other Opiliones (Figure S2a, Table S5). The relative abundance of Firmicutes in SOM, HOF, and WOC was 21.90%, 6.30% and 20.17% respectively, ranking second. Cluster analysis of symbiotic bacteria in each sample revealed that the species composition of Opiliones from Hainan Island and Beijing caves were more similar (Fig. 10a, b). Therefore, the study suggests that the environment of the caves in Beijing may be similar to that in Hainan Island, and provides insights into the origin of *S. martensi*.

The cave can be thought of as an independent “small world” with constant temperature and humidity, permanent darkness and limited food resources. Unlike terrestrial vertebrates, which have high diffusivity, terrestrial cave invertebrates are usually confined to small geographical areas and are the main components of cave fauna [40]. *S. martensi* has lived for a long time in isolated caves, with limited interaction with external biota, particularly from the south to the north. In addition, the northern climate is not fixed, with the onset of the monsoon climate from the Oligocene-Miocene boundary [84], East Asia has experienced seasonality of lower average temperature and humidity in winter [85]. Due to the relatively stable microclimate, caves may serve as a refuge for numerous organisms, and their surface counterparts tend to become extinct under adverse climatic conditions, which means some local cave biota remain [86]. This also explains why this study was unable to collect any other *Sinonychia* or even Laniatores closely related to *S. martensi* outside the cave from Beijing, only other harvestmen of the Eupnoi. A spider study suggested that climate change during the middle Miocene period facilitated the development of underground lifestyles in the middle latitudes of Asia [87]. The seasonality may have contributed to the extinction of surface species, and caves with relatively stable temperatures and humidity may have provided an ideal refuge for local species. In addition to climate change, human-made environmental destruction has impacted the distribution of *S. martensi*. With the decrease in temperature and humidity, reduction in surface trees, and destruction of the ecosystem, some Opiliones on the surface sought refuge underground and adapted to the stable cave environment, leading to changes in their morphology. With the passage of time and adaptation to the caves, their posture changed. The other part disappeared from the surface forever. Conversely, Laniatores, which are still found in the tropics and subtropics, have managed to survive until today.

## Conclusions

This study focused on the Opiliones in karst caves in Beijing, comparing them to those found outside the caves as well as to other organisms such as harvestmen, spiders and pseudoscorpions within the same cave. The results showed that the environment plays a more important role in determining symbiotic bacteria diversity than heredity. *S. martensi*, a unique species belonging to the Travunioidea and first discovered in China, adds to the mystery of caves that are both geographically isolated and have a unique environment. The symbiotic bacteria structure also reflects the characteristics of the host environment and the symbiotic community composition of Opiliones in caves in Beijing is similar to those found in Hainan Island, suggesting that the cave environment may resemble that found in the tropics. This finding explains the occurrence of *S. martensi* in caves in the north, and helps explore the role of symbiotic bacteria in host distribution and evolution, and in explaining the biological characteristics of the host.

### Electronic supplementary material

Below is the link to the electronic supplementary material.

Supplementary Material 1

Supplementary Material 2

Supplementary Material 3

Supplementary Material 4

Supplementary Material 5

Supplementary Material 6

Supplementary Material 7

## Data Availability

The sequence data generated and analyzed in this study are available at NCBI (https://www.ncbi.nlm.nih.gov) under accession numbers (PRJNA985231).

## Abbreviations

PCR: Polymerase Chain Reaction
ASVs: Amplicon Sequence Variants
PCoA: Principal Coordinate Analysis
NMDS: Non-Metric Multidimensional Scaling
