# Supplementary figures and images for: Environmental specificity of karst cave habitats evidenced by diverse symbiotic bacteria in Opiliones
Source: BMC Ecol Evol. 2024 May 8;24:58. doi: 10.1186/s12862-024-02248-9 (PMC11080181; doi:10.1186/s12862-024-02248-9)

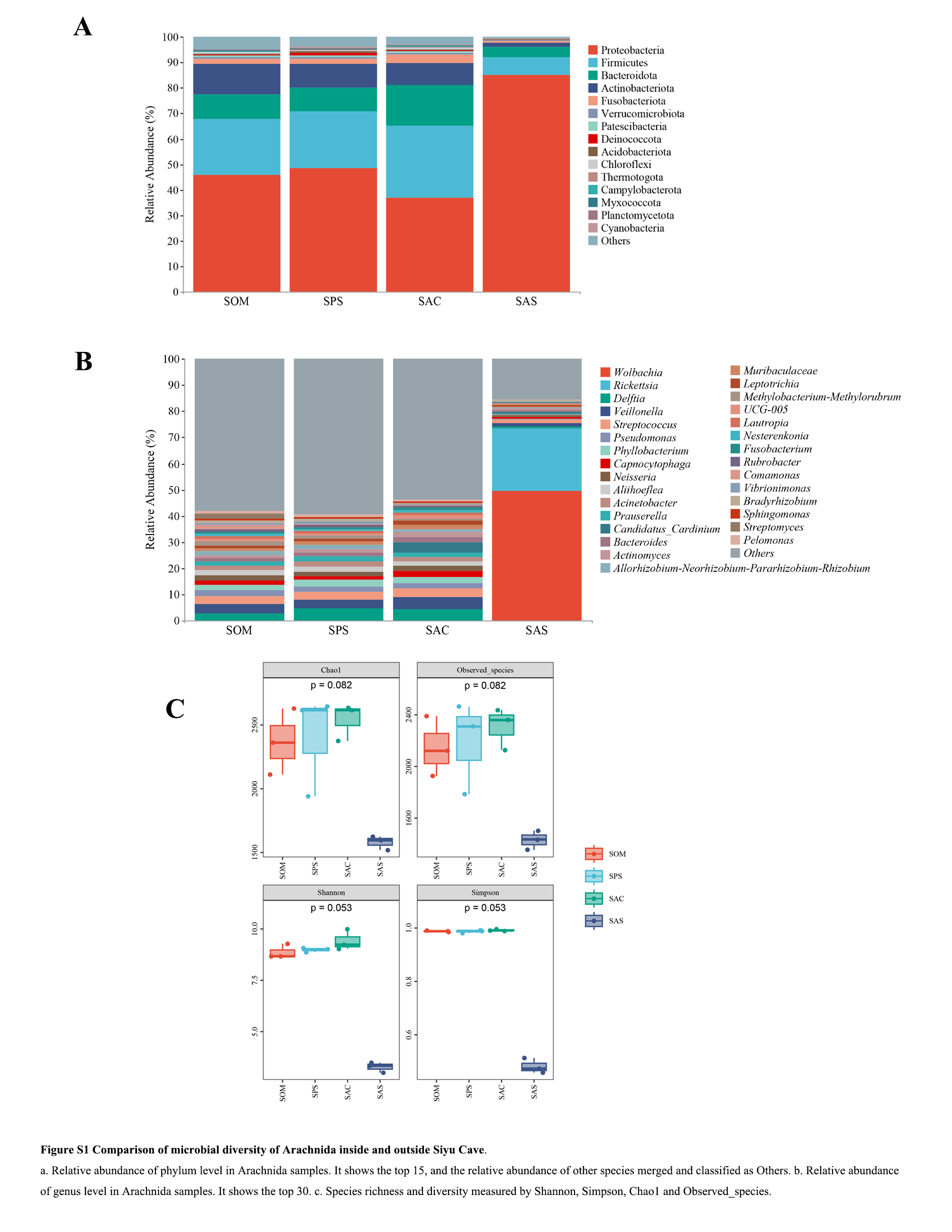

Supplement: Supplementary file 6 — Supplementary Material 6 [file 12862_2024_2248_MOESM6_ESM.tif]

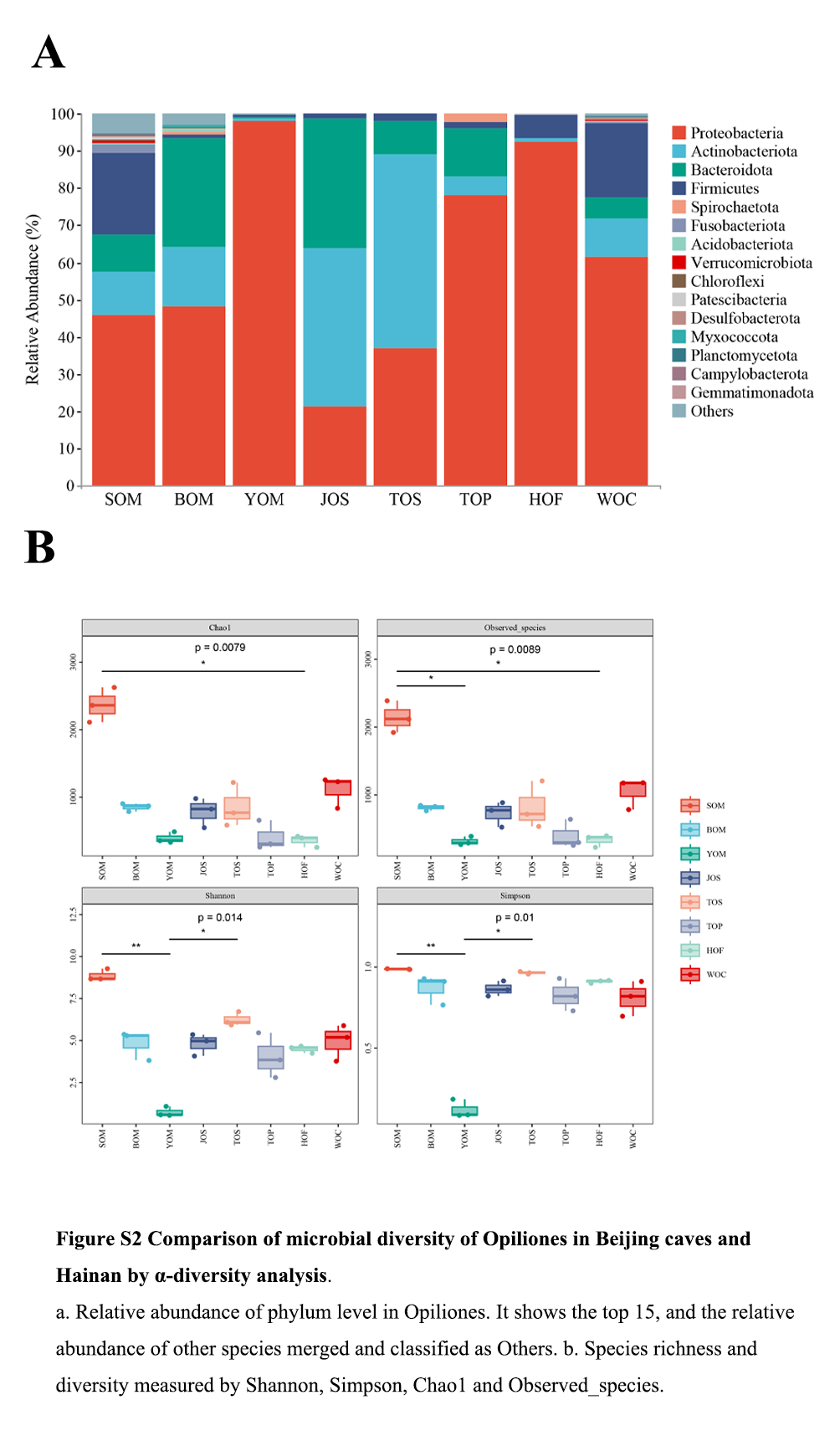

Supplement: Supplementary file 7 — Supplementary Material 7 [file 12862_2024_2248_MOESM7_ESM.tif]
